# Particle Manipulation in 2D Space Using a Capacitive Micromachined Ultrasonic Transducer

**DOI:** 10.3390/mi13040534

**Published:** 2022-03-29

**Authors:** Chang Hoon Lee, Beom Hoon Park, Young Hun Kim, Hyeong Geun Jo, Kwan Kyu Park

**Affiliations:** Department of Convergence Mechanical Engineering, Hanyang University, Seoul 04763, Korea; leechang93@hanyang.ac.kr (C.H.L.); pbh128@hanyang.ac.kr (B.H.P.); jason401@hanyang.ac.kr (Y.H.K.); sjdf5702@hanyang.ac.kr (H.G.J.)

**Keywords:** particle manipulation, CMUT, acoustic radiation pressure

## Abstract

Ultrasonic particle manipulation is a noncontact method for controlling microscale objects, such as cells or microparticles, using an acoustic field. In this study, a 2D array of capacitive micromachined ultrasonic transducers (CMUTs), placed horizontally in immersion, generated ultrasonic waves in the vertical direction, and the oil’s surface increased due to the radiation force of the ultrasonic waves. In addition, the radiation force directly exerted a force on a floating particle. By measuring the movement of the reflected laser light by the moving oil surface, the height of the oil’s surface deformed by the acoustic radiation force (ARF) was measured. The ARF made a floating particle, as well as the oil’s surface, move. The particle moved radially away from the surface position above the transducer, and its velocity was determined by its position on the fluid’s surface. When a single channel was operated, it moved 0.4 mm at an average speed of 90 μm/s, and when two adjacent channels were operated, it moved 1.2 mm at a speed of 272 μm/s. The particles moved in any direction on the surface of the oil by controlling the actuation channel using an electrical switch.

## 1. Introduction

Noncontact manipulation of microscale objects is required for research in biomedicine, chemistry, engineering, and physics; this includes the trapping, sorting, transportation, and positioning of particles. The study of microfluidic-based particle manipulation has been conducted using methods such as hydrodynamic, acoustic, electrical, magnetic methods, and optical tweezers [1]. Optical methods are mainly used to manipulate small particles of the nanometer size [2,3]. Optical tweezers were first proposed by Ashkin in 1970 [4,5]. These optical tweezers can manipulate dielectric particles with diameters in nanometers and micrometers. A single laser beam with a lens traps the particles in the beam. When particles in a medium exhibit a refractive index smaller than that of the particle with a focused laser, the particles are trapped at the point with the highest laser-light intensity by the gradient force. Ashkin used a 2.68 μm sphere which was drawn into the beam axis and trapped at the center of the argon laser beam with a radius equaling 6.2 μm and wavelength equaling 514.5 nm [4]. Additionally, two opposing equal Gaussian beams trapped a particle in a stable equilibrium at the symmetry point. The optical tweezers first proposed can be used for particles considerably smaller than the beamwidth of the laser and larger than the wavelength of the laser. Recent studies have shown that sub-wavelength particles can be manipulated by the laser [6]. A recently proposed method uses a slot waveguide to trap particles 10 nm in size. In contrast, the Bloch modes in a periodic structure can create potential wells that trap sub-wavelength particles. Optical tweezers have limitations in the size of particles that can be controlled because of the limitations of the beam width and the wavelength of the laser. However, the thermal effects of a high-focused laser light constitute a problem for biological applications [7].

Acoustic waves can manipulate particles of various sizes using different frequency values and produce fewer thermal effects compared to laser lights. Primitive acoustic tweezers have been developed based on the same principles used to develop optical tweezers [8]. Two focused lead zirconate titanate (PZT) transducers were used to generate a force potential well to trap a spherical particle. In reference, both focused transducers had a 3.5 MHz center frequency and were positioned facing each other. The transducers generated a potential well with a width proportional to the beam width to trap a particle. The trapped particle, with a 270 μm diameter, was smaller than the beamwidth of the transducers, as in the case of the optical tweezers. However, the particle size exceeded the wavelength of the transducers.

Apart from the primitive acoustic tweezer method, additional methods have been developed for the manipulation of particles of various sizes such as those based on standing waves [9,10,11,12], surface acoustic waves (SAWs) [13,14,15,16,17], and single-beam acoustic tweezers [16,17,18,19]. In the method based on acoustic standing waves, pressure nodes are utilized for particle manipulation. The primitive acoustic tweezer created a potential well using the symmetric arrangement of the same transducers. The standing-wave method of particle manipulation also uses several transducers. Transducers with an arranged symmetry created the standing wave field. Courtney et al. used four 5 MHz transducers as wave sources [9]. The counter-wave created a standing wave. The scattered particles were subsequently transported and captured at the node of the standing wave field. This method can generate different wavefields by controlling the phase of the wave [9]. In contrast, the two different material particles that were mixed in fluid flow were separated at the node and anti-node [11,12]. The transducers were attached to the side of the pipe containing fluid and different types of particles. Two particles were separated, and the node and anti-node were made by two opposing transducers.

The SAW method uses inter-digital transducers (IDTs). Orthogonal pairs of IDTs generate a standing SAW in microfluidic devices. The standing wave field can trap particles on the pressure node. By controlling the power input, the particle can be manipulated on the chip plane [13,14,15]. Single-beam acoustic tweezers (SBATs) use a focused ultrasound transducer. Gradient and scattering forces are generated when a particle encounters an ultrasound wave. When the particle size exceeds that of the wavelength of the wave, there is a point wherein the gradient force exceeds the scattering force. The net gradient and scattering forces trap a particle at the center of the beam [18,19,20,21]. An SBAT enables this trapped particle to move freely on a two-dimensional (2D) plane. Research has also been conducted using transducer arrays to manipulate a particle on a 2D plane without moving transducers [20]. A single channel of a capacitive micromachined ultrasound transducer (CMUT) was used as the single SBAT. Each channel of the CMUT created a pressure field with a fluid surface that generated a gradient force on the PDMS particles. The center frequency of the CMUT equaled 1.8 MHz, and the PDMS particles had a radius of 10 μm, which is considerably smaller than the wavelength.

Optical and acoustic manipulations have previously been conducted in fluids such as water. Additionally, the size of the particles is limited by the wavelength and beamwidth of the laser or sound waves. This suggests the existence of several limitations concerning the particle, such as those regarding its size or material, because of the property of the medium and the size of the laser (or transducer). Thus, research concerning the control of floating objects has been conducted. In the case of the optical method, photoresponsive surfactants cause dilatational flows at the liquid’s surface [22]. The flow patterns can be used to trap particles on the fluid at a lower intensity than conventional optical tweezers. Varanakkottu et al. used a wavelength of 325 nm and a wavelength of 442 nm to make the flow with surfactants [22]. The laser modified the surface tension of the fluid surface because of the photoresponsive surfactants that induced the flow of fluid on the surface to move the polystyrene particle with a radius of 15 μm. The particle direction could be changed by replacing the laser with one exhibiting a different wavelength.

The collection of particle clusters was studied using acoustic radiation forces [23]. The acoustic standing wave was created by a piezoelectric plate with a 938.5 kHz transducer. The fluid comprised a solution of 6 μm spherical copolymer particles in distilled water. By operating an acoustic standing wave within an open fluidic volume using a piezoelectric plate, the fluid profiles were changed, and the particles were clustered at the pressure anti-nodes. In contrast, objects could be transported via fluid flow [24]. Punzmann used a wave maker to generate surface waves. When the cylindrical wave generator created a wave at a constant frequency, the fluid flow occurred in a constant direction in which the particle moved [24]. If the wave generator frequency or amplitude of the plunger was changed, the wave direction also changed.

The transducers used in the standing wave method were located at the surface of the medium. A matching layer was required to match the acoustic impedance of the fluid with that of a sound-absorbing backing. Although the particles were controlled in the 2D plane by adjusting the phase differences of the respective pairs, the location of captured particles was restricted to the node or anti-node of the standing wave. The SAW method resembles the standing-wave method. The pair of transducers generated a standing wave field in the medium. The particles were captured at the node of the standing wave. The transportation of the particles is controlled via the frequency modulation (approximating the resonant frequency) of the two ultrasound transducers. The SBAT method is limited by particle size—the particle size should be smaller than the acoustic beam width. When the particles are transported by the transducer, they move to the spot where the particle is positioned. The system requires a mechanical motion system such as a linear stage.

In a previous study, we presented floating particles on the liquid that could move in one direction using ultrasound transducers in immersion. [25]. In this study, floating particles were manipulated in a 2D plane through an electrically controlled multiple transducers and feedback system. When the immersed transducer was employed to manipulate the particle, the matching layer to reduce the difference in acoustic impedance between the acoustic source and the target was not necessary. The electrical switch controlled each channel of the array such that the system was composed only of electrical parts. The transducer generated a pressure field that caused the acoustic radiation force to control the particle position on the oil’s surface.

## 2. Principle of Particle Manipulation

**Figure 1:**
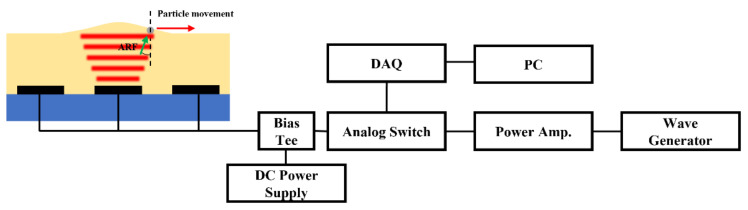
Schematic of particle manipulation principle and system.

This study proposes the manipulation of a particle on a fluid surface. As shown in Figure 1, a particle is present on a fluid (i.e., silicone oil) surface, and the device is actuated with a function generator. The ultrasound wave propagates upward and is subsequently reflected at the oil–air interface. The radiation pressure pushes the particle and the oil surface. As a result, the surface of the oil undergoes deformation, and the particle is pushed outward from the center of the transducer.

The acoustic radiation force is determined by the pressure field of the transducer. Each element of the CMUT used in this study is an 800 um square transducer with a center frequency of 2 MHz. The 3 dB angle of divergence of each element is 16 degrees, and the pitch between elements is 1 mm. For particle movement with this transducer array, the pressure fields of each transducer need to overlap. To satisfy this condition, it is necessary to satisfy the oil height of 1.7 mm or more. The height of the oil used in this paper was 2 mm.

The simulation was conducted using a commercial finite element analysis package (COMSOL Multiphysics, COMSOL Inc., Stockholm, Sweden) to calculate the pressure field and the radiation force at the air–oil interface. The physics used in the simulation was *pressure acoustics*. The results were analyzed in the frequency domain. The length and width of the domain were set to 5 mm, which is smaller than the actual experimental condition to reduce the computation time. Since the sidewall was a smaller domain than the real one, it was set as a radiation boundary condition in which the wave leaves the domain with minimal reflection. A single channel of the CMUT was assumed to represent a single square transducer. The size of the transducer in the simulation model was 800 μm, the same size as the single channel of the CMUT. The transducer was placed at the bottom of the oil, and the air was set above the oil. The size of the mesh was equal to one-fifth of the wavelength. The output pressure of the single transducer was calculated using the pressure sensitivity of the CMUT equaling 120 kPa with a frequency of 2 MHz, which was the same as the center frequency of the CMUT. The radiation force was calculated using the value of the pressure field. The radiation force was calculated using the following equations [26,27,28]:

(1a) $$F_{x}=-\frac{\rho_{0}}{2}\left(\frac{\partial Re\left[u_{x}^{2}\right]}{\partial x}+\frac{\partial Re\left[u_{x}u_{y}\right]}{\partial y}+\frac{\partial Re\left[u_{x}u_{z}\right]}{\partial z}\right)$$

(1b) $$F_{y}=-\frac{\rho_{0}}{2}\left(\frac{\partial Re\left[u_{y}u_{x}\right]}{\partial x}+\frac{\partial Re\left[u_{y}^{2}\right]}{\partial y}+\frac{\partial Re\left[u_{y}u_{z}\right]}{\partial z}\right)$$

(1c) $$F_{z}=-\frac{\rho_{0}}{2}\left(\frac{\partial Re\left[u_{z}u_{x}\right]}{\partial x}+\frac{\partial Re\left[u_{z}u_{y}\right]}{\partial y}+\frac{\partial Re\left[u_{z}^{2}\right]}{\partial z}\right)$$

The velocity of the particle was derived from the radiation force obtained through the Equation (1a–c) ($F_{x},\;F_{y}\;\mathrm{and}\;F_{z}$ is the Reynolds stress force, $\rho_{0}$ is medium density, and $u_{x},\;u_{y}\;\mathrm{and}\;u_{z}$ is the particle velocity). Radiation pressure pushed the particle radially away from the surface position above the transducer. The force of viscosity on a small sphere moving through a viscous fluid with a very small Reynolds number is represented using the Stokes drag force, which is given by $F_{d}=6\pi \mu rv$ (μ represents the dynamic viscosity, r is the radius of the spherical object, and v is the relative velocity between the object and flow). The velocity of the particle is determined using:

(2) $$ARF-F_{d}=m\frac{dv}{dt}$$

where ARF denotes the acoustic radiation force, $F_{d}$ is the drag force, m is the mass of the particle, and v is the velocity of the particle.

When the AC voltage was stopped to excite the CMUT, the fluid mound and radiation pressure diminished. Consequently, the particle stopped moving instantaneously because of the drag force, which had a very small time constant. It was possible to control the particle on the fluid’ surface by controlling each channel of the CMUT using an electrical switch.

## 3. Fabrication and Characterization of the CMUT

To deform the fluid’s surface, the CMUT can generate sufficient acoustic pressure. In addition, the array-type transducer can manipulate the particle on the 2D surface of the fluid. The characteristics of the CMUT were determined based on the cavity height, membrane thickness, and top-plate radius. To elevate the oil’s surface, the CMUT should generate adequate acoustic pressure. The CMUT designed herein had a thicker membrane and vacuum-gap height compared to those of the device used in ultrasonic imaging to achieve high sound pressure. The transducer was designed with a 115 μm radius for a center frequency of 2 MHz during immersion. Two fabrication processes were used to fabricate the CMUT: sacrificial release and wafer-bonding. The local oxidation of silicon (LOCOS) processes should be selected for thick gaps and membranes [29].

A 500 μm thick single-crystal silicon wafer was prepared as the substrate wafer. Thermal silicon oxide (SiO_2_), with a thickness of 100 nm, and stoichiometric silicon nitride (Si_3_N_4_), with a thickness of 200 nm, were deposited for the LOCOS process and insulation layer via low-pressure chemical vapor deposition (LPCVD). Thermal oxidation, which creates a vacuum gap with LOCOS, was conducted to achieve a target step height of 230 nm after dry etching of silicon nitride. Before the wafer bonding process, a chemical mechanical polishing (CMP) process is required, because the surface roughness of silicon oxide, which serves as the post of the CMUT, should have a minimal value (below 0.5 nm RMS) to guarantee the success of the fusion bonding.

**Figure 2:**
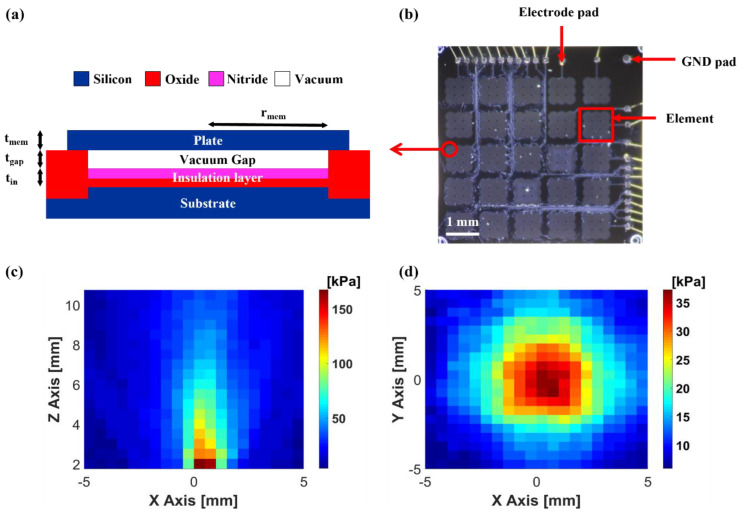
(**a**) Cross-section of the CMUT cell; (**b**) photograph of 2D CMUT array; (**c**) pressure field of the vertical direction; (**d**) pressure field of the horizontal direction at z = 11 mm.

A silicon-on-insulator (SOI) wafer with a 15 μm thick active layer of Si <1 0 0> was selected and bonded atop the substrate via fusion bonding and annealing at 1050 °C. The handling layer of the SOI wafer was wet-etched using tetramethylammonium hydroxide (TMAH) solution. The buried oxide layer was removed through buffered oxide etching (BOE), leaving the active layer of the plate covering the cavities after the layer served as an etch-stop layer for the TMAH etching process. Subsequently, dry etching was conducted to separate the CMUT elements. Furthermore, a portion of the opened LOCOS oxide layer was etched to expose the substrate silicon, which works as an electrical ground. An Al layer was deposited via metal sputtering to provide electrical connections to the top-most silicon plate and the substrate at the bottom. The wafer was diced after coating the photoresist to protect the device’s surface. Figure 2a depicts a schematic of the cross-section of the fabricated CMUT. A single device was composed of 25 channels placed in a 5 × 5 square layout, with a 1 mm pitch on a 6 × 6 mm die, as shown in Figure 2b. Each channel of the CMUT wass composed of nine elements with a 3 × 3 square arrangement and a size of 800 μm. The radius of the single element was 116 μm. The collapse voltage of the device was 115 V, and the center frequency was 2 MHz in oil with an 80 V bias voltage. The electrical connection between the CMUT and the power supply was established using a ceramic chip carrier with wire-bonding. A hydrophone (HNP-0400, ONDA Corp., Sunnyvale, CA, USA) was used to perform pressure measurements. All pressure values were measured at 50 V_pp_ AC voltage with 80 V DC bias voltage and calculated based on the sensitivity table of the hydrophone. The B-mode image in Figure 2c reveals that the pressure pattern was linear in the vertical direction. The C-mode image in Figure 2d depicts the horizontal plane of the pressure field 1.1 cm away from the device’s surface. The device had a 12 kPa/V surface pressure sensitivity that was computed from Figure 2c,d using the acoustic pressure loss via attenuation, diffraction, and a 3 dB spot, which was 3 mm in size.

## 4. Experimental Setup

In this study, the particle on the fluid’s surface was manipulated via fluid surface deformation and acoustic radiation pressure. When the transducer was operated by a long tone burst that could create a standing wave, the surface of the fluid was deformed by the acoustic radiation pressure. The deformed area was on the activated transducer and the surrounding area. When the particle was located on the slope of the deformed surface, it was transported by the acoustic radiation force exerted in a direction transverse to the acoustic beam. Depending on the position of the particle, it could be moved by changing the operating channel using 2D array transducers.

**Figure 3:**
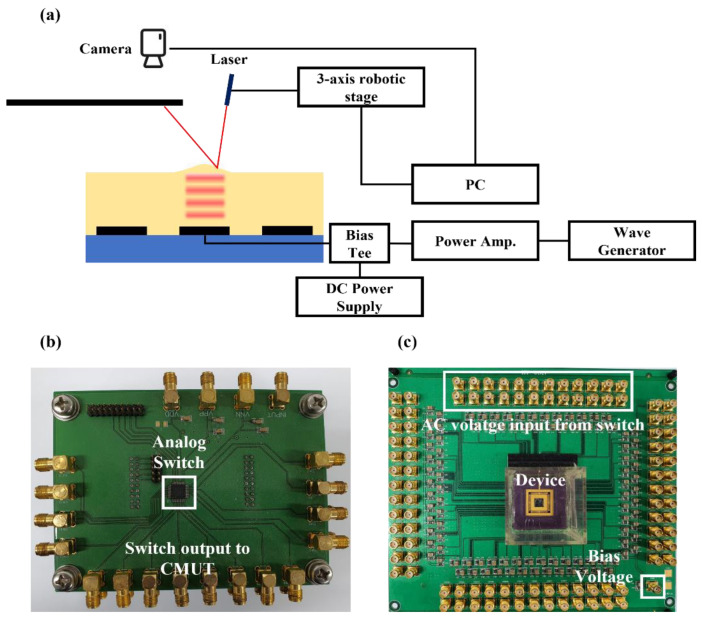
(**a**) Measurement system for the surface height of oil with a laser; (**b**) photograph of the channel-switching PCB board; (**c**) photograph of the CMUT-operating PCB board.

**Figure 4:**
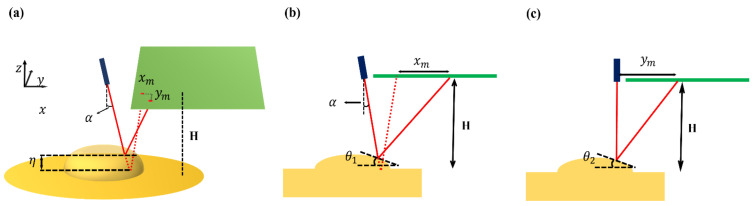
(**a**) Laser light reflection by moving oil surface; (**b**) XZ plane view; (**c**) YZ plane view.

The fluid surface deformation could be measured using a laser and camera. In Figure 3a, the laser light was incident on the surface of the liquid. The surface of the oil oscillated at the same frequency as the PRF, and the reflected laser light moved as the oil surface vibrated. The position data of this reflected laser light were obtained through the camera. The laser light in the video was imported into MATLAB on a frame-by-frame basis. The image of the laser spot was a circular shape. By acquiring and analyzing the image sequences, the center location of the laser spot could be calculated. The acquisition of the image and signal process was performed by MATLAB (MATLAB R2020a, The Math Works, Inc., Natick, MA, USA). The movement of the laser light was divided into vertical and horizontal movements. Since each element had a square shape, it was symmetrically identical in the height direction when the calculation was performed by scanning in the base direction. Figure 4 shows how the oil surface height by the movement of the reflected laser light was obtained. In Figure 4, $\theta_{1}$ is the angle of inclination between the *x*-axis and the oil surface, $\theta_{2}$ is the angle of inclination between the *y*-axis and the oil surface, H is the height between oil surface and laser pointer, $y_{m}$ is the displacement in the *y*-axis of the laser, $x_{m}$ is the displacement in the *x*-axis of the laser, α is the angle of incidence of the laser with respect to the surface, and η is the height of the oil surface. From Figure 4a–c, the height of the oil could be calculated by following Equations (3) and (4):

(3) $$\theta_{1}=\tan^{-1}(\frac{x_{m}}{H}+\tan\alpha )-\alpha$$

(4) $$\theta_{2}=\frac{1}{2}\tan^{-1}\frac{y_{m}}{H}$$

The surface height (η) is calculated by integrating the slope angle for each point with respect to the length.

The bias and operating voltages were 80 V DC and 20 V_pp_ AC, respectively, for the oil height measurement. The input voltage frequency was 2 MHz with 10 Hz pulse repetition frequency (PRF) and 50% duty cycles.

The radiation pressure should be sufficient to move the particle. The output pressure of the transducer was proportional to the input AC voltage. The input AC voltage was transmitted by a function generator with a power amplifier. The bias and operating voltages were 80 V DC and 50 V_pp_ AC, respectively, to increase the radiation pressure acting on the particle. The input voltage frequency was 2 MHz with 10 Hz PRF and 50% duty cycles. The experiments were recorded using a CMOS camera mounted on a microscope. The position of the sphere was tracked through a video recorded using MATLAB. The basic method used to identify the particle was the same as that used in the laser light track. The color of the particle in the video frame is written in MATLAB, which can write the movement of the particle in each frame. The velocity of the particle can be calculated based on the location of the particle using the frame rate of the recorded video.

The switching system consisted of an analog switch, a CMUT driving circuit board, and a digital data acquisition (DAQ) module, which is shown in Figure 3b,c. A high AC voltage was required to generate sufficient pressure to move the particle. A high-voltage analog switch (CPC7601, IXYS Corporation, Milpitas, CA, USA) was used to supply a high AC voltage to each channel of the CMUT. The logic voltage of the switch was controlled using a DAQ module (NI USB-6008, National Instruments Corporation, Austin, TX, USA). Each channel of the CMUT was controlled by an analog switch. This enabled the particle to move in a specific position at a specific speed.

**Figure 5:**
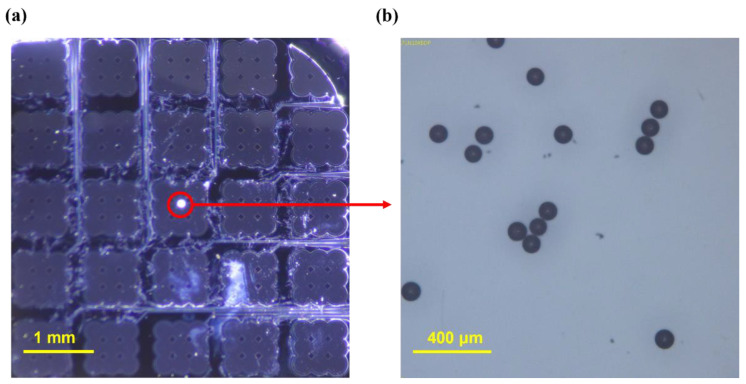
(**a**) Photograph of the floating particle captured via the device; (**b**) microscopic photograph of polyethylene particles.

A 2 mm thick silicone oil (AR 20) in a 2.5 × 2.5 cm oil tank, which was positioned at the center of the PCB board (Figure 3c), was employed as a medium, with a 1.01 g/mL density at 20 °C. As shown in Figure 5a,b, the particles were clear polyethylene microspheres (CPMS-0.96, 90–106 μm, Cospheric LLC, Goleta, CA, USA) with a radius in the range of 90–106 μm and a density of 0.96 g/cc, indicating that they were lighter than silicone oil.

## 5. Result and Discussion

**Figure 6:**
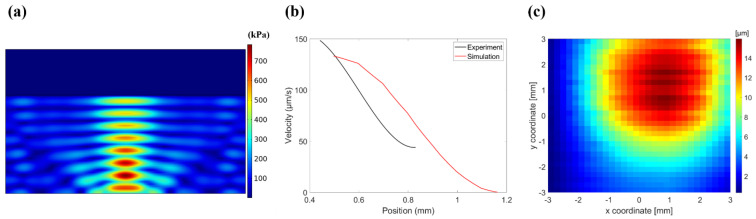
(**a**) Simulated pressure field in medium; (**b**) velocity comparison based on simulation and experiment locations; (**c**) measured oil height with laser pointer.

The simulation results depict the pressure field of the single channel, which was used to calculate the velocity according to the location. Figure 6a illustrates the simulated pressure field of the transducer. The surface pressure of the transducer was calculated using the pressure sensitivity of the CMUT. The number of burst cycles was sufficiently large to generate standing waves. Figure 6b shows the velocity at various locations. The particle floating on the off-axis position of the transducer was transported by acoustic radiation pressure.

The deformation of the oil’s surface indicates the profile of the acoustic radiation force. Figure 6c shows the measured height of the oil’s surface. The height of the fluid was 16 μm. The figure shows the area affected by the acoustic radiation force. If the particle was floating directly above the transducer, the particle remained stationary at the original location, because the radiation force was exerted in a direction orthogonal to the surface. When the particle was on the off-axis position of the transducer, the net radiation force was in the *x*-axis direction. When a single channel was operating, the particle stopped after 400 μm of movement from its initial position. However, the area of the deformed oil was much larger than the moving area. Errors can occur during measuring with a laser pointer owing to its spot size. Due to the difference in density between particles and oil, approximately 85% of the particles were immersed in oil. The volume immersed in the oil in the experiment was smaller than the volume immersed in the oil in the simulation. For this reason, the measured speed may be smaller than the result of the simulation.

**Figure 7:**
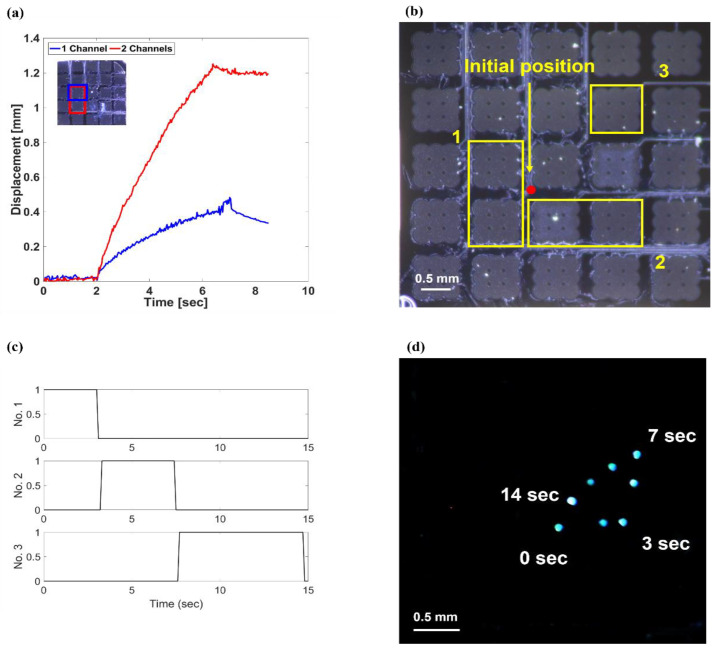
(**a**) Particle velocity operating on single and double channels; (**b**) operating channels for triangular manipulation; (**c**) input signal sequence for triangular manipulation; (**d**) stacked images of triangular manipulation.

The experiment was conducted to compare the velocities of the single-channel and double adjacent-channel operations. The velocity of the particle was derived from a video of the particle movement. The coordinates of the particle positioned concerning the original point were recorded frame by frame. As shown in Figure 7a, the device was activated at an instant between 2 and 6.4 s. The average speed of the particle equaled 90.9 μm/s when the single channel was actuated. When the two neighboring channels were activated, the average speed equaled 272.7 μm/s. A larger portion of the oil surface increased because of the superposition of the pressure fields when both channels operated. As the particle moved radially away from the actuating channels, the moving velocity decreased in both cases. The velocity reduced because of the drag force caused by the fluid viscosity. When the particle was far from its original position, the slope of the fluid mound decreased because of the radiation area; thus, the particle stopped moving. The initial speed in the one-channel operation was 128.5 μm/s. In contrast, the final speed was 27 μm/s. In the case of the two-channel operation, the initial speed was 382.5 μm/s, whereas the final speed was 150 μm/s. The calculated velocity was smaller than that obtained via the experiment. The drag force was calculated considering a completely submerged particle. Therefore, the drag force at the particle exceeded that reported in the experiment. In the range where the particle’s radius was less than its wavelength, the ARF was proportional to the submerged volume of the particle. As a result, the particle’s velocity was proportional to the square of the particle’s radius.

By controlling the active transducer with an analog switch, the locations of the particle throughout the device’s area could be controlled freely (Supplementary Materials Video S1). The experiment was conducted to move the particle in a triangular shape. As shown in Figure 7b–d, the particle moved in a triangular shape depending on the operating channel. In Figure 7b, the marked channels show the operating channel and order. Figure 7c shows the input signal sequence with the analog switch for triangular manipulation. The operating voltage was 50 V_pp_ AC with a DC bias voltage of 80 V. The frequency of the input voltage was 2 MHz with 10 Hz PRF and 50% duty cycles. Controlling the actuation channel was performed with a switch while checking the particle’s location through a CMOS camera. The first two channels were used to move the base line of the triangle. When the particle was moved to an appropriate position through the camera, the operating channel was changed to the two channels of number 2, and the particle was moved in the height line of the triangle. Finally, channel 3 was activated to make it move in the hypotenuse direction. Thus, it was possible to move the particle in any direction along the same plane. The velocities of the particle corresponded to those in the previous experimental results. The velocity of the particle depended on the angle of the surface and could be adjusted via acoustic pressure. The results demonstrate that the particle can be transported in any direction on the 2D plane with a full 2D array. In this experiment, the transducer had 25 channels with a size of 6 × 6 mm. The particle moved radially away from the surface position above the transducer. The direction of the movement of the particle was determined by the relative position of the particle and the transducer. If the transducer area is expanded by increasing the number of channels of the transducer, the particle can be controlled over a larger area.

To manipulate a particle floating on a liquid surface in real time, a system that moves the particle and a system that can change its direction in real time is required. A 2D CMUT array was used to move the particle. To quantitatively predict the speed and direction of particle movement, it is necessary to calculate the pressure field generated by the transducer and its ARF. The particle’s movement speed was measured and compared with the analyzed results when a single channel was operated. For real-time particle manipulation, the extent to which the particle moves when a single channel is activated must be known. The liquid’s surface was fluctuated by the acoustic waves. By recording the reflected laser light, the height of the oil’s surface was calculated. It showed the area of the single-channel pressure field. The velocity comparison of dual-channel and single-channel operation showed that the velocity of the particle can be controlled by determining the number of operating channels. When conducting an experiment, it is practically difficult to proceed with particles of various sizes. We performed the experiment with a single particle size due to the limited availability of microparticles with a low density of less than 1 kg/m^3^. The position of the particle was shown in real time through the camera. Depending on the real-time location, the electrical switch can change the operating channel to change the particle’s path in real time. The result of a moving particle in a triangular shape shows the possibility of moving in all directions in a 2D plane.

## 6. Conclusions

In this study, a particle floating on a fluid’s surface was manipulated with a 2D CMUT array. The radiation pressure from the acoustic standing wave raised the fluid surface. Laser light was used to measure the surface deformation. The movement of the laser light was recorded through a video. This video was used to recognize and analyze the pointer location. The movement of the laser light revealed the height of the surface deformation which, in turn, indicated the area of the pressure field. The particle could move on the free surface owing to the acoustic radiation force. It moved freely in a 2D plane using a full 2D ultrasound array with an electrical control system. In other words, the particle could be transported without a mechanical moving system. Additionally, the results show that it is possible to control the speed of a particle based on the number of operating channels. With a full 2D array and electrical switching system, the present research may be useful for biomedical applications involving the manipulation of a single particle in a 2D plane without direct contact.

## Supplementary Materials

The following supporting information can be downloaded at: https://www.mdpi.com/article/10.3390/mi13040534/s1, Video S1: Supplementary Video S1.
